# Successful Treatment with Bedtime Basal Insulin Added to Metformin without Weight Gain or Hypoglycaemia over Three Years

**DOI:** 10.3390/jcm9041153

**Published:** 2020-04-17

**Authors:** Bernardo Mertes, Sybille Gödde, Michael Piorkowski, Guido Kramer, Ulrich Alfons Müller, Nadine Kuniss

**Affiliations:** 1 Department for Diabetology, Centre for Cardioangiology Bethanien, AGAPLESION Hospital Bethanien, 60322 Frankfurt/Main, Germany; b.mertes@ccb.de (B.M.); s.goedde@ccb.de (S.G.); m.piorkowski@ccb.de (M.P.); 2 Endocrinology and Metabolic Diseases, Department of Internal Medicine III, Jena University Hospital, 07740 Jena, Germany; guido.kramer@med.uni-jena.de; 3 Practice for Endocrinology and Diabetology, Centre for Ambulatory Medicine, Jena University Hospital, 07743 Jena, Germany; UA.Mueller@med.uni-jena.de; 4 Outpatient Healthcare Centre Dr. Med. Kielstein, 99096 Erfurt, Germany

**Keywords:** basal insulin, NPH insulin, hypoglycaemia, metformin

## Abstract

The aim of this observational study was to follow-up patients with bedtime basal insulin (NPH insulin) added to metformin. In 285 patients with type 2 diabetes, a therapy with bedtime basal insulin added to metformin was started due to failure to achieve a glycaemic goal. Up until July 2019, 272 patients (95.4%) were followed-up (59.5 y, 92.6 kg, diabetes duration 6.6 y, HbA1c 8.4%/68.6 mmol/mol). HbA1c decreased by −1.2% and bodyweight by −1.7 kg after a duration of 31.7 ± 29.1 (range 2–133) months. Severe hypoglycaemia did not occur. In 144/272 patients (52.9%), the therapeutic goal for HbA1c was achieved over 32.7 months. In 69/272 patients (25.4%), the HbA1c target was achieved over 25.0 months (afterwards, therapy with basal insulin was discontinued because HbA1c was under target). In 36/272 patients (13.2%), the HbA1c goal was achieved until the submission of this manuscript (mean duration of treatment 57.4 ± 28.2 (range 13–121) months). Over 90% of patients with type 2 diabetes and failure of metformin reached their HbA1c goal with additional basal insulin at bedtime over several years in association with a reduction of bodyweight and without any event of severe hypoglycaemia.

## 1. Introduction

After the failure of comprehensive lifestyle management and patient education, metformin is the first-line drug therapy in patients with diabetes mellitus type 2 [1,2]. But despite intensive diabetes management, after several years, some patients do not meet the individual glycaemic targets with metformin alone.

If the metabolic control deteriorates and a refresher course in patient education fails, adding a second oral hypoglycaemic agent is a common strategy to improve metabolic control [3]. However, one of several options recommended in the German guidelines for the treatment of patients with diabetes type 2 is adding basal insulin at bedtime to metformin (basal supported oral therapy, BOT) [1]. Furthermore, the “German Association of General Practitioners” suggests adding basal insulin at bedtime after treatment failure of metformin and glibenclamide [4].

The German Federal Joint Committee (GBA) recommends human insulin as the first choice. However, long-acting insulin analogues (e.g., glargine) are widely used in Germany [5]. To start, the “German Association of General Practitioners” recommends 8–16 international units (IU) per day [4]. There could be advantages by treatment with an intermediate-acting basal insulin overnight. Injected in the evening, this insulin only works until the next morning. Because intermediate-acting basal insulin works for 8 to 12 h, the patients treated in this way do not receive insulin during the day. Therefore, counting of blood sugar components according to carbohydrate units, blood glucose tests during the day, multiple insulin injections per day as well as insulin dose adjustments are not necessary. Additionally, the risk of hypoglycaemia is lower compared with intensive insulin therapy with multiple injections of short-acting insulin [6]. Furthermore, Bergenstal et al. recommended bedtime insulin to initiate insulin therapy as a part of a systematic management plan for people with type 2 diabetes and inadequate glycaemic control [7]. Several trials investigated the efficacy and safety of metformin in combination with intermediate-acting insulin [8,9,10,11,12,13,14]. However, evidence for long-term effect of “basal supported oral therapy” up to “treatment failure” (i.e., failure to achieve individual treatment goals for glycaemia) is not available.

Therefore, the aim of this longitudinal, observational study was to follow-up patients with bedtime intermediate-acting (basal) insulin (NPH-insulin) added to metformin with special focus on glycaemic control, bodyweight, frequency of severe hypoglycaemia, insulin dose and time within individual treatment goals.

## 2. Methods

### 2.1. Participants and Study Design

Compliance with ethical standards. Ethical standard: The study was conducted in accordance with the ethical standards laid down in an appropriate version of the 1964 Declaration of Helsinki. Human and animal rights: All procedures followed were in accordance with the ethical standards of the responsible committee on human experimentation (institutional and national) and with the Helsinki Declaration of 1975, as revised in 2008 (5). The study was approved by the Ethics Committee of the “State Chamber of Physicians of Hesse” (number 2019-1261-evBO, date of approval 11 October 2019).

**Figure 1:**
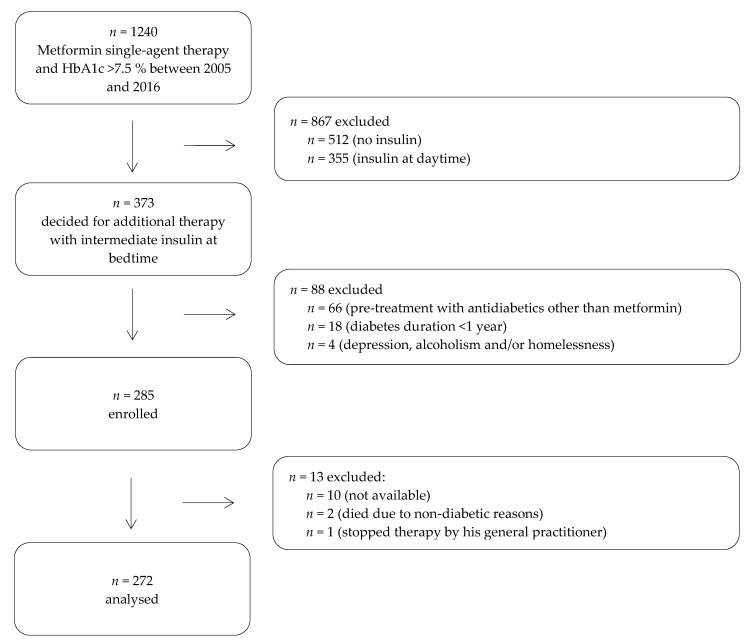
Study Design.

Between 2005 and 2016, 1240 patients with type 2 diabetes and metformin single-agent therapy presented in an outpatient centre for diabetology at secondary care level (Figure 1). All patients had missed their individual therapy target for HbA1c with monotherapy of metformin (HbA1c > 7.5%). After explaining the different therapy options (a combination of oral hypoglycaemic drugs, incretins or insulin), 373 patients decided on therapy with intermediate-acting (basal) insulin (NPH-insulin) at night continuing metformin. Of 373 patients, 88 were excluded for various reasons (see Figure 1), while 285 were able to be enrolled in our study.

In order to create a homogeneous cohort, we included only patients with the following criteria: clinically confirmed diabetes mellitus type 2 treated with metformin single-agent therapy for at least one year, non-achievement of the therapeutic goal for HbA1c and cognitive ability to carry out the insulin therapy independently. Furthermore, we declared the following exclusion criteria: pre-treatment with hypoglycaemic drugs other than metformin, contraindications for the continuation of treatment with metformin (e.g., renal failure with glomerular filtrate rate detected less than 30 mL/min, catabolism, NYHA 4 heart failure or metformin intolerance), consuming diseases and life expectancy less than four years, corticosteroid therapy, severe cognitive or psychiatric disorders, alcoholism, homelessness and pregnancy. Until 31 July 2019, 272 of 285 patients (95.4%) were re-examined and analysed retrospectively. Two patients died due to non-diabetic reasons, one patient has stopped therapy (and has been adjusted to a combination of hypoglycaemic drugs by his general practitioner) and 10 individuals were not available. For patient characteristics at baseline see Table 1. There were no differences between study participants (*n* = 272) and drop-out (*n* = 13).

### 2.2. Treatment

All patients were recommended to take part in a target group-specific, structured treatment and teaching programme (STTP) adapted from the education programme of Grüßer and Jörgens [15]. The content of the adapted STTP includes: insulin types, insulin injection, self-measuring of blood glucose, documentation, management of hypoglycaemia. Additionally, nutrition and exercise were important contents of patient education. The patients were given a realistic target of weight loss: 2 to 3 kg within 3 to 6 months.

Therapy with basal insulin at night was started with 8 IU. Basal insulin should be injected subcutaneously into the thigh immediately before bedtime. The dose determined by the physician should not be changed. Under no circumstances should the basal insulin be injected during the day.

At the beginning of insulin therapy, all patients were instructed to measure blood glucose at 10 p.m., 2 a.m., 5 a.m. and 7 a.m. for two consecutive nights and to document the results in a specially developed protocol sheet. The protocol sheet was then examined by a physician and the insulin dose adjusted to suit the patient’s needs. This was done to prevent night-time hypoglycaemia. For reasons of economy and to reduce the risk of changes in insulin dose by the patient, daily self-monitoring of blood glucose was expressly discouraged. Patients should continue their dietary habits and metformin therapy unchanged.

The patients visited the medical practice for follow-up every 3 to 6 months. At each visit, HbA1c was measured. If the individual target value for HbA1c was missed, the insulin dose was adjusted. If the HbA1c was below the individual target, the dose was reduced by 2 IU. At a dose of 4 IU, therapy with basal insulin was terminated. If the value was above the target, the insulin dose was increased by 2 to 4 IU. After each change in insulin dose, the patient was asked to measure and document blood glucose at 10 p.m., 2 a.m., 5 a.m. and 7 a.m. for two consecutive nights and to submit the protocol sheet to the physician. The insulin dose was left if blood glucose values had remained stable over the night. Therapy with basal insulin was terminated when a nightly drop in blood glucose was recorded while the target value for HbA1c was missed. Duration of successful treatment was defined when the last targeted HbA1c was measured.

### 2.3. Outcomes

Clinical and routine laboratory data were recorded at baseline and at each visit, respectively, and documented in the electronic patient record. Baseline characteristics were collected at the time of the first injection of basal insulin.

HbA1c was measured using “Adams A1c HA-8180V” with a normal range of 4.5 to 6.1%.

Severe hypoglycaemia was defined as a condition with the necessity of glucagon injection (administered by a third party, e.g., relatives) or intravenous glucose injection (administered by medical professionals) with or without hospitalisation according to the guidelines of the German Diabetes Association [16]. Frequency of non-severe hypoglycaemia was not determined.

### 2.4. Statistical Analysis

All continuous data are presented as mean ± standard deviation (SD). Categorical data are described by absolute and relative frequencies. To compare two groups, unpaired *t*-test was used for continuous variables and Fisher’s exact test was performed for categorical variables. Paired *t*-test was used regarding the difference between baseline and follow-up in both groups. To compare more than two groups, univariate ANOVA was used. Multiple linear regression was used to determine which factors lead to the longest possible therapeutic success with the combination of metformin and bedtime basal insulin. Duration of treatment (months) was determined as a dependent variable. Assumed variables were age, duration of diabetes, duration of metformin therapy, change of weight, baseline BMI, baseline HbA1c and change of HbA1c. Additionally, the successful therapy with bedtime basal insulin and metformin is shown in a Kaplan-Meier curve (Kaplan-Meier curves are defined as the probability of being free from an event in a given length of time).

Significance was defined at the 0.05 level. An HbA1c change was considered as clinically relevant if the value changed by at least 0.5%. Statistical analysis was performed using the Statistical Package IBM SPSS Statistics 25 (IBM Corp. Released 2017. IBM SPSS Statistics for Windows, Version 25.0. Armonk, NY, USA).

## 3. Results

Successful treatment with NPH insulin and metformin was reached over a mean duration of 31.7 ± 29.1 months: Range 2 to 133 months (median duration: 22.0 months). In all participants, HbA1c decreased from 8.4 ± 0.9% (68.6 ± 9.6 mmol/mol) to 7.2 ± 0.8% (54.7 ± 8.3 mmol/mol; *p* < 0.001, Table 2). Furthermore, bodyweight decreased by −1.7 kg (baseline 92.6 ± 18.5 kg, follow-up 90.9 ± 18.4 kg, *p* < 0.001). Severe hypoglycaemic events did not occur in any patient.

Overall, the duration of successful treatment with the combination of metformin and bedtime basal insulin was very different between the patients. Afterwards, we created four groups to describe the different results of the patients (characteristics of each group are shown in Table 1, changes of HbA1c and weight in Table 2):

Group A “treatment failure after short duration” (≤9 months): In 23 of 272 patients (8.5%), the therapeutic goal for HbA1c was not achieved after a mean duration of 4.5 ± 1.6 months (range 2–9 months) (Table 2) despite adjustment of insulin dose to 14.1 ± 5.7 IU. The combination of metformin and basal insulin at bedtime had to be supplemented by a third hypoglycaemic drug or intensification of insulin therapy (i.e., switch to twice daily mixed insulin or prandial insulin) was necessary.

Group B “treatment failure after long duration” (>9 months): In 144 of 272 patients (52.9%), the therapeutic goal for HbA1c was achieved over 32.7 ± 28.5 months. Mean insulin dose was 14.8 ± 4.9 IU. After 32.7 months a third antidiabetic agent was added or switch to conventional/intensified insulin therapy was necessary to reach HbA1c target.

Group C “treatment failure because of overtreatment”: In 69 of 272 patients (25.4%), the therapeutic goal for HbA1c was achieved over 25.0 ± 24.4 months. After 25.0 months, basal insulin at night was discontinued because HbA1c was <6.5%. Subsequently, metformin was again prescribed as monotherapy. Additionally, weight was reduced from baseline to follow-up by −4 kg (over a 25 month period).

Group D “ongoing treatment”: In 36 of 272 patients (13.2%), the therapeutic goal for HbA1c was achieved up to the 31 July 2019. After a mean duration of 57.4 ± 28.2 months (range 13–121 months), the combination of metformin and bedtime basal insulin is successfully performed. HbA1c decreased from 8.5 to 7.2%, weight from 91.0 to 88.4 kg (Table 2). Mean insulin dose was 14.4 ± 5 IU.

**Figure 2:**
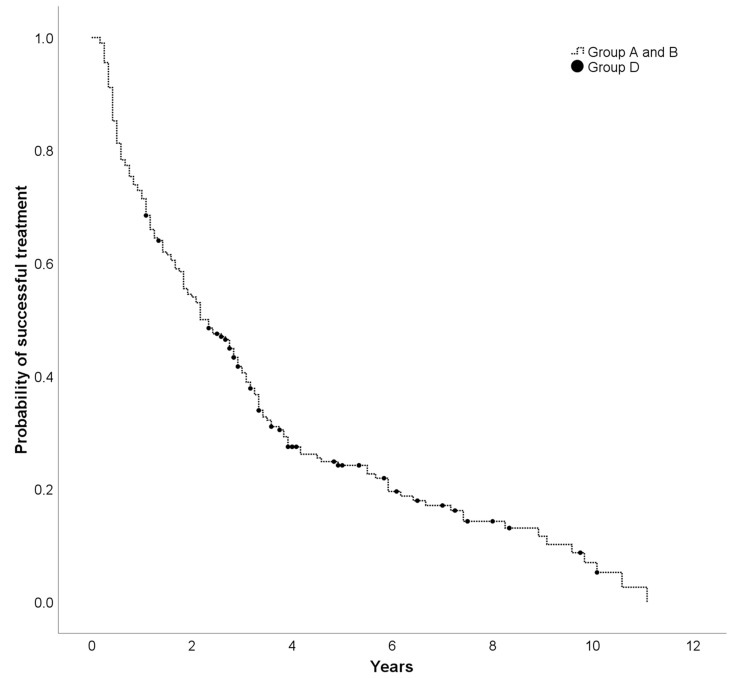
Successful therapy with bedtime NPH-insulin and metformin (Group A, B and D).

In summary, 91.5% of the participants benefited from the combination of metformin and basal insulin (Group B, C and D). 8.5% had no advantage (Group A). Patients of Group A had a significantly higher baseline BMI than individuals in Group B, C and D (Table 1, other variables were not different between the groups). Successful therapy with bedtime basal insulin and metformin of Group A, B and D over the years is shown in a Kaplan-Meier curve (Figure 2). After adjustment for age, duration of diabetes and metformin therapy, baseline BMI and HbA1c as well as a change of weight and HbA1c, multiple linear regression showed that weight loss leads to the longest possible therapeutic success with the combination of metformin and bedtime basal insulin (*p* < 0.001).

Figure 2 does not contain Group C because basal insulin was stopped due to overtreatment not due to treatment failure.

## 4. Discussion

After the failure of metformin monotherapy, over 90% of patients with type 2 diabetes reached their individual HbA1c goal with additional application of NPH insulin without any event of severe hypoglycaemia over several years. About half of the patients maintained this diabetes therapy over 2.6 years before their diabetes therapy had to be intensified (Group B) and an additional 13% reach their individual HbA1c target by the manuscript submission (Group D).

Furthermore, 25% could stop their insulin therapy after two years (Group C). We can only speculate as to the reasons, which may include eating habits. Maybe, these patients have changed their living habits and reduced their bodyweight. These patients lost 4 kg over two years. Moreover, weight reduction was associated with the longest duration of treatment with basal insulin. This may be due to the absence of insulin administered during the day.

Only 8.5% of the participants had no benefit from adding basal insulin to metformin (Group A). After only 4.5 months, intensification of diabetes therapy with a third antidiabetic agent or switch to twice daily mixed insulin or prandial insulin was necessary. The reason could be due to the baseline weight. Patients of Group A had a higher BMI at baseline than people who benefit from a combination of metformin and basal insulin.

After the failure of metformin, a possible option is adding basal insulin at bedtime recommended in the guidelines [1,4] because treatment with basal insulin at night can have some advantages. Adherence to a diet plan with a classification of blood sugar components according to carbohydrate units, multiple daily insulin injections and insulin dose adjustments, as well as self-monitoring of blood glucose, is not necessary. If basal insulin does not reach the HbA1c target at night, switching to other insulin therapies (conventional or preparandial insulin therapy) is easier because patients have already learned how to handle insulin.

Furthermore, intermediate basal insulin injected in the evening only works until the next morning (in contrast, analogue insulin works for up to 24 h). In conclusion, the risk of hypoglycaemia is lower with once-daily basal insulin than conventional or intensified insulin therapy [17]. There is no difference in costs of analogue insulin compared to human insulin for patients in the statutory health insurance in Germany but costs are up to 10-fold higher in other countries [18]. During our study, no event of severe hypoglycaemia occurred in any patient. A reason may be due to the careful increase of basal dose as well as the relatively low insulin dose used to treat the cohort. In two guidelines where basal insulin at night is explicitly recommended as the first-choice treatment for initial insulin adjustment, an initial dose of 8 to 16 IU [4] or 18 IU [19] is recommended. In our study, insulin therapy was started with 8 IU and increased to a mean of 13.4 IU after nightly blood glucose monitoring. By structured measurement of blood glucose during the night, the prescription of a too high insulin dose could be avoided. By measuring blood glucose in the morning alone, as recommended in the guideline by Permanente et al. [19], hypoglycaemia during night-time cannot be detected.

In contrast to our study with an HbA1c reduction of 1.2% and a weight reduction of 1.7 kg with basal supported oral therapy (insulin dose of 13 IU), after a mean follow-up of 2.6 years, Holman et al. showed in the 4T-Study [6] an opposing outcome in patients with metformin and sulfonylurea and addition of bedtime long-acting insulin analogue detemir (Table 3). The same HbA1c reduction of 1.2% was achieved by a significantly higher daily insulin dose and a weight gain of 3.6 kg after 3 years. Furthermore, the median rate of severe hypoglycaemia was in 1.7 per patient per year but not in any patients in our study. Possible reasons could be the structure of care, i.e., general practitioner, diabetologist, hospital outpatient department, and the participation in a STTP.

The APOLLO-Study with bedtime insulin glargine showed no severe hypoglycaemic event and an HbA1c reduction of 1.7%, but a weight gain of 3 kg in 44 weeks [8]. Philis-Tsimikas showed an HbA1c reduction of 1.7% and a weight gain of 1.6 kg with 33 IU evening basal human insulin and an HbA1c reduction of 1.5% and a weight gain of 0.7 kg and with 37 IU evening detemir, respectively [9].

Furthermore, there are some other studies showing that the combination of bedtime insulin plus metformin is superior against other therapy options [10,11,12]. The study by Gerstein et al. showed that adding long-acting insulin to oral hypoglycaemic drugs is more likely to achieve a lower HbA1c level than therapy with oral agents [10]. In comparison to different combinations of metformin, glyburide and bedtime insulin, metformin plus bedtime insulin seems superior with respect to improvement in glycaemic control, frequency of hypoglycaemia and weight gain [11]. Equally, the Cochrane review by Vos et al. showed that addition of metformin to insulin has positive effects on glycaemic control and insulin requirements in people with type 2 diabetes and inadequate glycaemic control compared to other oral agents [12].

The trial by Raskin et al. showed an HbA1c reduction of 2.4% and weight gain of 3.5 kg with bedtime long-acting insulin (glargine) plus metformin and other drugs, respectively, after 28 weeks [13]. However, 51 IU was needed to achieve a plasma glucose target between 80 and 110 mg/dl (4.4 and 6.1 mmol/L). In addition, baseline HbA1c was higher (9.8%) than in our study. The study by Civera et al. showed a comparably high baseline of 9.6%. However, HbA1c decreased only by 0.7% with a weight gain of 1.7% over 24 weeks [14].

Overall, previous studies (Table 3) found significant weight gain, whereas our study showed weight reduction after metformin and intermediate basal insulin. There are many possible causes, which unfortunately cannot be explained from our data. In addition to weight loss, the following reasons can be considered: daily calorie intake, amount of carbohydrates, exercise, stress, insulin resistance, residual insulin, genetic factors, social status, etc. The patients were given a realistic target of weight loss: 2–3 kg within 3 to 6 months.

A further reason might be patient education [20]. However, none of these studies [6,8,9,10,11,12,13,14] contains information about self-monitoring of glucose or patient training. That is a strength of our study. Furthermore, the present study is a longitudinal investigation with a long follow-up over the years. However, there are limitations, i.e., the lack of a control group. Furthermore, the frequency of non-severe hypoglycaemia was not recorded.

## 5. Conclusions

In an outpatient cohort, over 90% of patients with type 2 diabetes and failure of metformin monotherapy reached their individual HbA1c goal with additional basal insulin at bedtime over several years in association with a reduction of bodyweight as well and without any event of severe hypoglycaemia.

## Figures and Tables

**Table 1:** Baseline characteristics of study participants.

Parameter	All (*n* = 272)	Drop Out (*n* = 13)	Group A (*n* = 23)	Group B (*n* = 144)	Group C (*n* = 69)	Group D (*n* = 36)
Age (years)	59.5 ± 11.0	56.8 ± 7.7	59.3 ± 10.2	58.9 ± 10.8	59.3 ± 11.6	62.6 ± 11.0
Female *n* (%)	95 (34.9)	5 (38.5)	10 (43.5)	51 (35.4)	21 (30.4)	13 (36.1)
Diabetes duration (years)	6.6 ± 4.7	5.4 ± 4.4	5.6 ± 4.7	6.6 ± 4.2	6.7 ± 5.1	7.4 ± 5.8
Treatment duration with metformin (years)	4.8 ± 3.8	3.8 ± 2.5	4.0 ± 4.4	4.8 ± 3.5	5.1 ± 3.9	4.8 ± 4.1
Previous participation in a STTP *n* (%)	220 (80.9)	8 (61.5)	20 (87.0)	118 (81.9)	53 (76.8)	29 (80.6)
BMI (kg/m^2^)	31.6 ± 5.8	31.3 ± 5.1	34.6 ± 6.9 ^†^	31.1 ± 5.2 ^†^	32.4 ± 6.8 ^†^	30.2 ± 4.3 ^†^
Bodyweight (kg)	92.6 ± 18.5	87.7 ± 17.2	98.7 ± 24.3	91.0 ± 17.4	95.0 ± 20.7	91.0 ± 13.4
HbA_1c_ (%)	8.4 ± 0.9	8.8 ± 0.7	8.5 ± 1.0	8.5 ± 0.9	8.2 ± 0.8	8.5 ± 0.9
HbA_1c_ (mmol/mol)	68.6 ± 9.6	72.3 ± 7.7	69.8 ± 11.0	69.3 ± 9.8	66.3 ± 8.5	69.3 ± 9.9
eGFR (ml/min)	120.6 ± 41.2	120.6 ± 33.9	135.7 ± 48.3	118.1 ± 39.3	122.0 ± 46.1	118.3 ± 32.6
Concomitant diseases *n* (%)						
Neuropathy	96 (35.3)	4 (30.8)	9 (39.1)	50 (34.7)	23 (33.3)	14 (38.9)
Retinopathy	9 (3.3)	0 (0)	0 (0.0)	3 (2.1)	6 (8.7)	0 (0.0)
Hypertension	240 (88.2)	11 (84.6)	21 (91.3)	127 (88.2)	62 (89.9)	30 (83.3)
Coronary heart disease	67 (24.6)	3 (23.1)	6 (26.1)	34 (23.6)	17 (24.6)	10 (27.8)
Peripheral arterial disease	21 (7.7)	0 (0)	1 (4.3)	12 (8.3)	4 (5.8)	4 (11.1)
Myocardial infarction	21 (7.7)	1 (7.7)	1 (4.3)	13 (9.0)	4 (5.8)	3 (8.3)
Apoplex	7 (2.6)	0 (0)	1 (4.3)	5 (3.5)	1 (1.4)	0 (0.0)

Abbreviations: BMI = Body Mass Index, eGFR = estimated Glomerular Filtration Rate, IU = international units, STTP = structured treatment and teaching programme. ^†^ *p* < 0.05 (univariate ANOVA).

**Table 2:** Changes in HbA1c and weight.

Participants	HbA1c (%)		Bodyweight (kg)	
	Baseline	Follow-Up	Baseline	Follow-Up
All (*n* = 272)	8.4 ± 0.9	7.2 ± 0.8 ^†^	92.6 ± 18.5	90.9 ± 18.4 ^†^
Group A (*n* = 23, 4.5 months)	8.5 ± 1.0	8.5 ± 0.8	98.7 ± 24.3	97.8 ± 23.8
Group B (*n* = 144, 32.7 months)	8.5 ± 0.9	7.3 ± 0.4 ^†^	91.0 ± 17.4	90.4 ± 17.2
Group C (*n* = 69, 25.0 months)	8.2 ± 0.8	6.3 ± 0.5 ^†^	95.0 ± 20.7	91.0 ± 20.8 ^†^
Group D (*n* = 36, 57.4 months)	8.5 ± 0.9	7.2 ± 0.4 ^†^	91.0 ± 13.4	88.4 ± 13.1 ^†^

^†^*p*-value <0.05 between baseline and follow-up.

**Table 3:** Comparison of studies with basal supported oral therapy.

Study	Intervention (years)	Duration	HbA1c (%)		Weight (kg)	Insulin Dose (IU)	Severe Hypoglycaemia
			Baseline	Change			
Present study	bedtime intermediate acting insulin (human) plus metformin	2 months –11 years	8.4	−1.2	−1.7	13	0
Holman et al. 2009 [6]	bedtime basal insulin (detemir) plus metformin/sulfonylurea	3 years	8.1	−1.2	+3.6	88 *	1.7/patient/year **
Bretzel et al. 2008 [8]	bedtime long-acting insulin (glargine) plus oral agents	44 weeks	8.7	−1.7	+3.0	42	0.03/patient/year **
Philis-Tsimikas et al. 2006 [9]	evening intermediate acting insulin (human) plus oral drugs	20 weeks	9.2	−1.7	+1.6	33	0 **
	evening basal insulin (detemir) plus oral drugs		8.9	−1.5	+0.7	37	2 cases in 169 patients **
Gerstein et al. 2006 [10]	bedtime long-acting insulin (glargine) plus oral agents	24 weeks	8.6	−1.6	+1.9	38	1 case in 206 patients **
Yki-Jarvinen et al. 1999 [11]	bedtime intermediate acting insulin (human) plus metformin and glyburide	52 weeks	9.9	−2.1	+1.2	20	0 **
	bedtime intermediate acting insulin (human) plus glyburide		9.8	−1.8	+3.9	24	0 **
	bedtime intermediate acting insulin (human) plus metformin		9.8	−2.5	+0.9	36	0 **
Raskin et al. 2005 [13]	bedtime long-acting insulin (glargine) plus metformin/other drugs	28 weeks	9.8	−2.4	+3.5	51	1 case in 1 patient **
Civera et al. 2008 [14]	bedtime intermediate acting insulin (human) plus metformin	24 weeks	9.6	−0.7	+1.7	21	0 **

Abbreviations: IU = international units. * 81.6% of the patients taking two types of insulin after 3 years. ** Hypoglycaemic events requiring third party assistance.

## Abbreviations

BMI	Body Mass Index
HbA1c	Glycated haemoglobin
IU	international units
NPH	Neural Protamine Hagedorn
STTP	structured treatment and teaching programme
